# Extraction of American ginseng polysaccharide by ultrasound-assisted deep eutectic solvents-based three-phase partitioning: Process optimization, structural characterization, and anti-ulcerative colitis study

**DOI:** 10.1016/j.ultsonch.2024.107206

**Published:** 2024-12-16

**Authors:** Zhongnan Wu, Chong Li, Junhao Li, Tanggan Wang, Meifeng Li, Leyi Zhao, Huimei Ye, Jiaheng Chen, Jiajia Zan, Lijun Song, Qian Zhang, Shaojie Zhang

**Affiliations:** a The Affiliated Dongguan Songshan Lake Central Hospital, Guangdong Provincial Key Laboratory of Research and Development of Natural Drugs, School of Pharmacy, Guangdong Medical University, Dongguan 523808, China; b School of Pharmacy, Guangdong Provincial Key Laboratory of Advanced Drug Delivery, Guangdong Provincial Engineering Center of Topical Precise Drug Delivery System, Guangdong Pharmaceutical University, Guangzhou 510006, China; c College of Life Sciences, Shandong Agricultural University, Tai’an 271018, China; d CAS Key Laboratory of Regenerative Biology, Joint School of Life Sciences, Guangzhou Institutes of Biomedicine and Health, Chinese Academy of Sciences, Guangzhou, Guangdong 510530, China

**Keywords:** Deep eutectic solvents, Ultrasonic, American ginseng, Polysaccharide, *Drosophila*, Anti-ulcerative colitis activity

## Abstract

Three-phase partitioning (TPP) is promising for isolating bioactive polysaccharides, but *t*-butanol’s environmental impact limits its application. Deep eutectic solvents can serve as a green and recyclable alternative to *t*-butanol. This study introduces an ultrasonic-assisted DES three-phase partitioning (UA-TPP-DES) system to extract and purify American ginseng polysaccharides (AGPs). The optimized DES-4, composed of lauric acid and nonanoic acid (molar ratio = 1:1), achieved a 35.28 % extraction yield under specific conditions and can be recycled five times with minimal yield loss. AGP-DES-4 has a broad molecular weight distribution (2.48–174.64 kDa) and mainly consists of mannose, glucose, galactose, and arabinose. *In vivo Drosophila* models show that AGP-DES-4 improves UC fly survival and enhances intestinal barrier function by regulating the proliferation and differentiation of intestinal epithelial cells (IECs) and intestinal stem cells (ISCs). Our results highlight the effectiveness of the AGP-DES-4 extraction method and its potential therapeutic value for treating UC.

## Highlights

• DES is well-suited as green solvents, replacing *t*-butanol in TPP system.
• UA-TPP-DES system develop to extract and purify American ginseng polysaccharides.
• UC *Drosophila* model is used to evaluate the anti-UC activity of AGP-DES-4.
• AGP-DES-4 show anti-UC activity by regulating the homeostasis of IECs and ISCs.

## Graphical abstract

**Figure:**
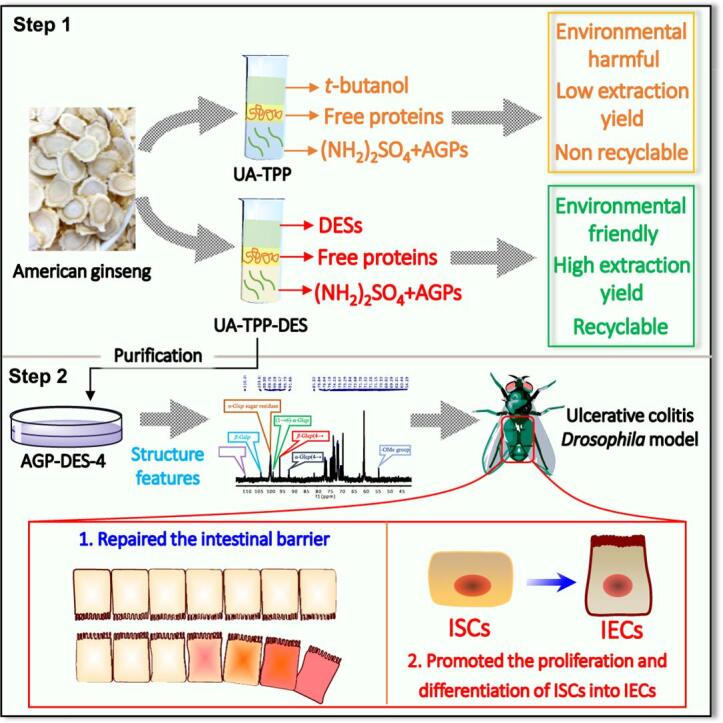

## 1 Introduction

American ginseng (*Panax quinquefolius L.*), an araliaceous perennial of the Panax genus, is cultivated worldwide. It originally grew in Quebec, Canada, and Wisconsin, and was successfully introduced to China in the 1980s. Today, it is widely cultivated in Northeast China, including Liaoning, Heilongjiang, and Jilin provinces [1]. American ginseng has broad applications in biomedicine and health products due to its notable pharmacological activities, including anti-tumor effects, cardiovascular protection, anti-oxidation, and anti-inflammatory properties, which are closely related to its active compounds, such as saponins, peptides, essential oils, and polysaccharides [2]. Among these components, ginsenosides have long been regarded as the primary contributors to its pharmacological effects. However, recent research increasingly highlights that polysaccharides play a significant role in the medical benefits of American ginseng by effectively enhancing immunity, inhibiting tumor growth, lowering blood glucose levels, and repairing intestinal damage [3]. Despite these promising findings, American ginseng polysaccharides (AGPs) have often been undervalued and discarded in recent decades due to their complex structural characteristics and the mistaken belief that they are not easily absorbed by the human body. This has led to significant resource waste. Therefore, developing efficient methods to extract AGPs and investigating their bioactivities are crucial for the optimal utilization of American ginseng resources.

Extraction technology plays a crucial role in the exploration, development, and application of natural polysaccharides. The hot water extraction (HWE) method is a green, economical, and effective way to extract polysaccharides [4]. However, the disadvantage of low extraction efficiency significantly limited the development of HWE. With advancements in technology, the extraction methods of polysaccharides are constantly improving and innovating, and more and more auxiliary extraction methods, such as ultrasound [5], microwave [6], enzyme [7], and others [8], are being applied in the extraction of bioactive polysaccharides, addressing the challenges of low efficiency. In addition to these methods, dilute acid/base or organic solvent was also used for polysaccharide extraction to achieve the aim of increasing yield. However, these approaches can alter the structure and activity of the polysaccharides [9], and may pose risks to personnel and the environment [10]. Among these methods, enzyme-assisted extraction methods are relatively milder and more environmentally friendly than acid/base or organic solvent extraction methods. Nevertheless, challenges such as enzyme residues and instability continue to hinder its application [11]. Therefore, it remains crucial to search for efficient and green extraction methods for natural polysaccharides.

Three-phase partitioning (TPP), an efficient bio-separation method, is receiving extensive attention. This method involves adding extra salt and an organic solvent to the raw extract, forming a three-layered phase distribution system. Low molecular weight substances, such as pigments and lipids, primarily dissolve in the top (organic) phase, while most proteins settle in the middle phase. Highly polar substances, on the other hand, are mainly found in the bottom (aqueous) phase [12]. Due to its high efficiency and convenience, TPP is commonly used for extracting and purifying polysaccharides and proteins [13], [14]. Although TPP offers numerous advantages over traditional extraction techniques, it also presents some drawbacks in practical applications. The use of volatile, combustible, and toxic *t*-butanol is a key challenge in TPP operations. Therefore, finding alternative, less toxic solvents is of great significance.

Deep eutectic solvents (DESs), composed of two or more components (one hydrogen bond acceptor and one or more hydrogen bond donors), are intentionally created through techniques such as heated stirring, grinding, freeze-drying, *etc.* [15]. DESs are reported to have several advantages over conventional solvents, including straightforward synthesis, eco-friendliness, low toxicity or non-toxicity, and promising recyclability [16]. Due to their environmentally friendly properties, DESs are potential substitutes for conventional organic solvents in extraction and isolation procedures [17]. DESs have been effectively used for extracting various bioactive compounds, such as flavonoids [18], alkaloids [19], and polysaccharides [20]. TPP is an efficient and convenient method for polysaccharide extraction and separation, but the volatility, toxicity, and difficulty in recovering organic solvents like *t*-butanol limit its practical application. The green, non-toxic, and recyclable characteristic of DESs enhances the feasibility of using TPP in the extraction and isolation of natural active components. Therefore, the strategic integration of TPP and DESs is proposed as an innovative method for isolating and purifying natural bioactive ingredients, including polysaccharides.

Model organisms are crucial in the study of disease pathogenesis as well as in the evaluation and screening of drug activity. Traditional high-throughput drug screening methods typically rely on *in vitro* cell models, biochemical analyses, or receptor binding analyses [21]. Though *in vitro* cell models are commonly employed for drug screening, their toxicity outcomes often fail to fully replicate tissue-specific reactions in the human body. In addition to cell models, mammals are frequently used in drug screening. However, their use is often limited by long experimental cycles, high costs, and reproductive and ethical constraints. Recently, *Drosophila* melanogaster has proven to be an excellent model organism for studying disease pathogenesis and drug screening. Its advantages include small size, rapid reproduction, short life cycle, simple physiological structure, low culture cost, clear genetic background, and ease of genetic manipulation. Moreover, *Drosophila* exhibits significant homology with human organs and genes [22]. These characteristics make *Drosophila* a valuable tool in modern research, including studies on the mechanisms of ulcerative colitis (UC) prevention and treatment, as well as drug screening.

Thus, to further develop AGP resources and explore a more efficient TPP system, this study aimed to establish an ultrasound-assisted DES-based TPP system for extracting and purifying polysaccharides from the roots of American ginseng. Single factor assays and Box-Behnken design (BBD) were employed to optimize the AGP extraction process. The structural features of the obtained AGP (labeled as AGP-DES-4) were further analyzed through molecular weight determination, monosaccharide composition analysis, Fourier transform infrared (FT–IR) spectroscopy, and nuclear magnetic resonance (NMR) spectroscopy. Finally, *Drosophila melanogaster* was used as a model organism to assess the beneficial effects of AGP-DES-4 on DSS-induced intestinal injury *in vivo*.

## 2 Materials and methods

### 2.1 Materials and reagents

Roots of American ginseng (origin Canada, batch number: 231003) were obtained from Guangdong Kangzhou Pharmaceutical Co., Ltd (Guangdong, China) and identified by Dr. Liangyun Zhou, Guangdong Pharmaceutical University (Guangzhou, China). The sample of American ginseng is stored in the Guangdong Provincial Key Laboratory of Research and Development of Natural Drugs, Guangdong Medical University, with reference number 20231024. Choline chloride, 1,6-Hexanediol, decanoic acid, lauric acid, terpineol, nonanoic acid, and octanoic acid were sourced from Shanghai Macklin Biochemical Co., Ltd. (Shanghai, China). Monosaccharide standards (glucose, galactose, xylose, arabinose, rhamnose, mannose, galacturonic acid, and glucuronic acid) were purchased from Shanghai Yuanye Bio-Technology Co., Ltd. (Shanghai, China). 1-Phenyl-3-methyl-5-pyrazolone (PMP) and dextran sulfate sodium (DSS) were obtained from Aladdin Bio-Chem Technology Co. Ltd. (Shanghai, China). Isoflurane was sourced from Suzhou Ruigu Medical Technology Co., Ltd (Suzhou, China). Paraformaldehyde (PFA) was obtained from Labgic Technology Co., Ltd. (Beijing, China), while the DAPI and 7-AAD staining reagent were from Servicebio Technology Co., Ltd. (Wuhan, China) and Abcam (Shanghai, China), respectively. All reagents utilized in this research were analytical grade.

### 2.2 Ultrasonic-assisted three-phase partitioning (UA-TPP) for AGP extraction

UA-TPP was used to produce American ginseng polysaccharide (AGP-TPP). The extraction method followed a previously reported procedure with minor modifications [23]. In brief, the powdered dried roots of American ginseng (0.1 g) were immersed in a 25 % wt (NH_2_)_2_SO_4_ solution at a 1:20 solid-to-liquid ratio. Subsequently, 2 mL of *t*-butanol was added to the mixture, and the extraction was carried out at 60 °C under ultrasonic conditions (100 W) for 30 min. Following extraction, the sample was centrifuged at 4000 rpm for 10 min to yield the bottom fraction. This fraction was then concentrated and further purified through dialysis against tap water for 48 h. Dialysate was processed via freeze-drying to obtain AGP-TPP. The yield of AGP-TPP was determined using the phenol–sulfuric acid method and a glucose standard curve.

### 2.3 Ultrasound-assisted deep eutectic solvents-based three-phase partitioning (UA-TPP-DES) for AGP extraction

#### 2.3.1 Preparation of DESs

The DESs used for polysaccharide extraction were prepared as follows. In brief, the hydrogen bond acceptor (HBA) and hydrogen bond donor (HBD) were carefully combined in a round-bottom flask, and subsequently subjected to ultrasonication at 70 °C until a clear and uniform solution was achieved. Detailed DES composition is elucidated in Table 1.

**Table 1:** DESs with different compositions.

No.	Solvent composition	Molar ratio
DES-1	Choline chloride: 1,6-Hexanediol	1: 2
DES-2	Decanoic acid: Lauric acid	1: 1
DES-3	Lauric acid: Terpineol	1: 2
DES-4	Lauric acid: Nonanoic acid	1: 1
DES-5	Lauric acid: Octanoic acid	1: 1

#### 2.3.2 Optimization of DES solvent for AGP extraction

0.1 g of American ginseng root powder was mixed with different DESs (DES-1, DES-2, DES-3, DES-4, and DES-5) at a solid-to-liquid ratio of 1:20. Then, 2 mL of 25 % wt ammonium sulfate was added to the solution to form the three-phase partitioning (TPP) system. The extraction was performed under the following conditions: temperature of 60 °C, moisture content of 30 %, ultrasonic power of 100 W, and ultrasound time of 30 min. Following extraction, the sample was centrifuged at 4000 rpm for 10 min. The lower layer was collected, concentrated, and then subjected to 48 h of dialysis in flowing tap water. The dialysate was then freeze-dried. The polysaccharides extracted using DES-1 to DES-5 were labeled as AGP-DES-1 to AGP-DES-5, respectively. The yields of the AGP-DESs were determined using the phenol–sulfuric acid method and the glucose standard curve.

### 2.4 Single factor experiment design for optimal conditions

Under fixed ultrasonic settings, variables affecting ultrasonic-assisted DES extraction include extraction duration and temperature, ammonium sulfate content, liquid–solid ratio, and the volume ratio of the upper and lower phases. To establish the optimal conditions for ultrasonic-assisted DES extraction of AGP, the effects of extraction time (10 min, 20 min, 30 min, 40 min, and 50 min), extraction temperature (30 °C, 40 °C, 50 °C, 60 °C, and 70 °C), ammonium sulfate mass fraction (10 %, 15 %, 20 %, 25 %, and 30 % wt), liquid–solid ratio (1:10, 1:20, 1:30, 1:40, and 1:50), and volume ratio of the top phase to the bottom phase (1:0.25, 1:0.5, 1:1, 1:1.5, 1:2, and 1:2.5) on the yield of AGP-DES-4 were investigated using single factor experiments. Based on the findings of single-factor experiments, three key influencing factors, namely the liquid–solid ratio (A), the volume ratio of the top phase to the bottom phase (B), and the ammonium sulfate mass fraction (C), were selected as independent variables at three levels (1, 0, −1) for the Box-Behnken design (BBD). A total of 17 groups of experiments were conducted, with the yield of AGP-DES-4 as the evaluation indicator. The three factors, three level experimental design were demonstrated in Table 2, and each group was randomly tested during the experimental period. In addition, the optimal extraction process was determined via regression analysis, and the validation tests were conducted under the identified conditions to verify the accuracy of the experiment.

**Table 2:** Factors and levels for Box Behnken center combination experimental design.

Levels	Factors		
	A	B	C
	Liquid-solid ratio	Volume ratio of the top phase to the bottom phase	Mass fraction of (NH_4_)_2_SO_4_
−1	1: 10	1: 0.25	5 %
0	1: 20	1: 0.5	10 %
1	1: 30	1: 1	15 %

### 2.5 Recycling and reusing of DES

The recycling and reusing experiment was conducted to evaluate the reusability of the DES solvent after AGP extraction. AGP-DES-4 was extracted using the optimal process described in Section 2.4. The extraction efficiency of AGP-DES-4 was assessed by comparing the yields from the initial extraction with those from subsequent extractions.

### 2.6 Structural characterization

#### 2.6.1 Relative molecular weight distribution

The relative molecular weight distribution of AGP-DES-4 was evaluated via HPGPC, a technique used in our previous study [24]. The detailed experiment procedures were shown in the Supplementary Materials S1.

#### 2.6.2 FT–IR analysis

The FT–IR spectrum of AGP-DES-4 were recorded using Shimadzu IR Affinity-1 spectrometer in the ranges of 200–400 nm and 4000–400 cm^−1^, respectively.

#### 2.6.3 Monosaccharide composition

The monosaccharide composition of AGP-DES-4 was established via the PMP pre column technique, as detailed in our previous report [25]. The detailed experiment procedures were shown in the Supplementary Materials S2. In addition, the quantification of the monosaccharides in AGP-DES-4 was further determined using the same method with glucose, mannose, arabinose, and galactose as the standards.

#### 2.6.4 NMR analysis

AGP-DES-4 (50 mg) was dissolved in 500 μL of deuterium oxide for NMR analysis. The ^1^H and ^13^C NMR spectra of AGP-1, acquired on a Bruker AV-500 instrument, were interpreted via MestReNova software (Version 14.0.2, Mestrelab Research SL, Spain).

#### 2.6.5 Congo red analysis

The Congo red assay was used to detect the presence of triple-helical structure in AGP-DES-4. In brief, the AGP-DES-4 solution (0.5 mg/mL) was thoroughly mixed with Congo red solution (50 μmol/L) in a concentration gradient of NaOH solution (0.00–0.60 M). Then, each mixed solution was scanned with a UV–Vis spectrophotometer in the range of 400–600 nm. The maximum absorption wavelength (λmax) of the sample was compared with the λmax absorption of Congo red in NaOH solution determined by the same method.

### 2.7 *In vivo* anti-UC evaluation

#### 2.7.1 *Drosophila* strain and maintenance

*W^1118^* wild type and *esg*-Gal4, USA-GFP/Cyo transgenic *Drosophila* melanogaster were gifted from Prof. Qian Zhang (School of Pharmacy, Guangdong Pharmaceutical University). The flies were maintained in a constant temperature and humidity incubator with temperature and humidity set as 25 °C and 65 %, under a 12 h light/12 h dark cycle.

#### 2.7.2 Establishment UC *Drosophila* model and of survival assay

*W^1118^* type of *Drosophila* that had emerged for 3–5 days were collected and randomly divided into control group (NC group), model group (DSS group), and AGP-DES-4 administration groups, with 30 flies (15 male and 15 female) in each group. Before the experiment, the flies were subjected to a 2-hour starvation period. After that, each group of *Drosophila* was transferred into an empty culture tube containing three filter paper soaked in 5 % sucrose, 3 % DSS, and different concentrations of AGP-DES-4. Especially, the filter papers in NC group were infiltrated with 5 % sucrose solution, the filter papers in DSS group were infiltrated with 3 % DSS + 5 % sucrose solution, while the AGP-DES-4 treatment groups were infiltrated with 1 % AGP-DES-4, 5 % AGP-DES-4, 10 % AGP-DES-4 (each containing 5 % sucrose + 3 % DSS), respectively. The total experimental period was 7 days, and the filter paper was exchanged every 24 h. During the experiment, the survival rate of *Drosophila* in each group was recorded and calculated. The formula for calculating survival rate is as follows.

$$Survival\;rate\;\left(\%\right)=\frac{Number\;of\;surviving\;flies\;per\;time\;point}{Total\;number\;of\;flies\;at\;the\;beginning\;of\;the\;experiment}$$

#### 2.7.3 Intestinal morphology assay

The female flies (*W^1118^* type) that had emerged for 4–5 days were randomly divided into 3 groups, which were NC group (containing 5 % sucrose), DSS group (containing 5 % sucrose and 3 % DSS), and 5 % AGP-DES-4 treated group (containing 5 % sucrose, 3 % DSS, and 5 % AGP-DES-4). Flies were undergoing a 2 h starvation treatment before being subpackaged into the empty culture tube, which contained 3 pieces of round filter papers (containing 5 % sucrose in NC group; 5 % sucrose + 3 % DSS in DSS group; 5 % sucrose + 3 % DSS + 5 % AGP-DES-4 in AGP-DES-4 treated group). The experiment lasted for 4 days, with the filter papers replaced daily. At the end of the experiment, the flies in each group were anesthetized with isoflurane, and the intestines of the flies in each group were collected. The collected intestines were sealed with 78 % glycerol and then imaged using a microscope. The intestinal length was measured and quantified using ImageJ software.

#### 2.7.4 Blue dye feeding assay

The blue dye feeding assay was used to evaluate the integrity of the intestines. In brief, the edible blue dye was diluted into the concentration of 2.5 % (w/v) using 5 % of sucrose solution. The blue dye was purified using a 0.22 μm filter, then preserved under sterile conditions for subsequent testing. Three stock solution were further prepared using 2.5 % of edible blue dye (diluted with 5 % of sucrose solution) as follows.

NC solution: 2.5 % of edible blue dye (diluted with 5 % of sucrose solution).
DSS solution: DSS diluted into the concentration of 4 % (w/v) using NC solution.
AGP-DES-4 solution: AGP-DES-4 diluted into the concentration of 4 % (w/v) using DSS solution.

The female flies (*W^1118^* type) that had emerged for 3–4 days were randomly divided into 3 groups, which were NC group (treated with 200 μL of NC solution), DSS group (treated with 200 μL of DSS solution), and 5 % AGP-DES-4 treated group (treated with 200 μL of AGP-DES-4 solution). The total experimental time was 7 days with the filter papers refreshed daily. After the experiment, the flies in each group were anesthetized with isoflurane and imaged using a stereomicroscope. After imaging, the flies were crushed and homogenized using phosphate buffer, and the absorbance was detected at 632 nm to evaluate the degree of blue dye immersion in flies.

#### 2.7.5 Effect of AGP-DES-4 on the intestinal precursor cell of *Drosophila*

Female *esg*-Gal4, USA-GFP/Cyo transgenic flies were randomly divided into three groups (30 flies/each group), including NC group, DSS group, and 5 % AGP-DES-4 treated group. The flies in each group were treated with DSS or 5 % AGP-DES-4 according to the procedures described in Section 2.7.3. At the end of the experiment, the intestines of the flies were collected and washed twice with PBS solution, followed by fixation in 4 % paraformaldehyde for 20 min. After fixation, the intestines were further washed with PBS three cycles to remove the residual fixatives and impurities. The samples were then stained with DAPI solution (50 μL) for 10 min under dark conditions. After staining, the intestines were washed twice with PBS to remove the unreacted DAPI. Finally, the obtained samples were sealed with 78 % glycerol and examined using a fluorescence microscope. ImageJ software was utilized to assess the fluorescence intensity.

#### 2.7.6 Effect of AGP-DES-4 on the intestinal epithelial cell of *Drosophila*

Female *W^1118^* type flies were randomly divided into three groups (30 flies/each group) including NC group, DSS group, and 5 % AGP-DES-4 treated group. The drug treatment methods were the same as those detailed in Section 2.7.5. Following drug treatment, the intestines of the flies were collected and washed twice with PBS. They were then incubated with 50 μL of 7-AAD solution (5 μg/mL) for 30 min in the dark. After staining, the samples were further washed three times with PBS to remove the excess 7-AAD solution. Subsequently, 200 μL of 4 % paraformaldehyde solution was used to fix the samples. After fixation, the samples were centrifuged at 500 rpm for 2 min to remove residual paraformaldehyde solution. The samples were then washed twice with PBS, followed by the addition of 50 μL DAPI and a 10-minute incubation in the dark. After incubation, the samples were washed twice with PBS to remove the unreacted DAPI solution. Finally, the samples were sealed with 78 % glycerol and inspected using a fluorescence microscope. ImageJ software was utilized to assess the fluorescence intensity.

### 2.8 Statistical analysis

Data was reported as means ± SD, and all extractions and fly trials were conducted with a minimum of three replicates. Group variances were analyzed using one-way ANOVA with multiple comparisons in GraphPad Prism 8.0. Statistical significance was set at *P* < 0.05 for ANOVA results, with significance levels indicated as *P < 0.05, ^**^P < 0.01, ^***^P < 0.001.

## 3 Results and discussion

### 3.1 DES screening and selection for AGP extraction

**Fig. 1:**
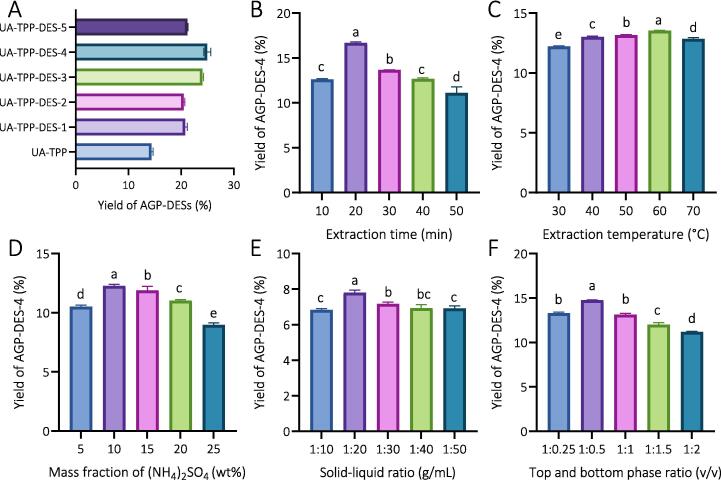
DES screening and selection for AGP extraction. (A) UA-TPP and UA-TPP-DESs extraction system on the yield of AGP-DES-4. (B) Effect of extraction time on the yield of AGP-DES-4 based on UA-TPP-DES-4 extraction system. Condition: extraction temperature: 60 °C, ultrasound power: 100 W, solid–liquid ratio: 1: 20 g/mL, mass fraction of (NH_4_)_2_ SO_4_: 25 % wt, volume ratio of the top phase to the bottom phase: 1: 1. (C) Effect of extraction temperature on the yield of AGP-DES-4 based on UA-TPP-DES-4 extraction system. Condition: extraction time: 20 min, ultrasound power: 100 W, solid–liquid ratio: 1: 20 g/mL, mass fraction of (NH_4_)_2_ SO_4_: 25 % wt, volume ratio of the top phase to the bottom phase: 1: 1. (D) Effect of mass fraction of (NH_4_)_2_ SO_4_ on the yield of AGP-DES-4 based on UA-TPP-DES-4 extraction system. Condition: extraction temperature: 60 °C, extraction time: 20 min, ultrasound power: 100 W, solid–liquid ratio: 1: 20 g/mL, volume ratio of the top phase to the bottom phase: 1: 1. (E) Effect of solid–liquid ratio on the yield of AGP-DES-4 based on UA-TPP-DES-4 extraction system. Condition: extraction temperature: 60 °C, extraction time: 20 min, ultrasound power: 100 W, mass fraction of (NH_4_)_2_ SO_4_: 10 % wt, volume ratio of the top phase to the bottom phase: 1: 1. (F) Effect of upper and lower phase ratio on the yield of AGP-DES-4 based on UA-TPP-DES-4 extraction system. Condition: extraction temperature: 60 °C, extraction time: 20 min, ultrasound power: 100 W, mass fraction of (NH_4_)_2_ SO_4_: 10 % wt, solid–liquid ratio: 1: 20 g/mL. Different letters indicate the statistical significance at *P* < 0.05.

Previous studies have demonstrated that DES composition significantly impacts the efficiency of plant polysaccharide extraction [26]. Thus, to search for a suitable DES solvent to form the TPP system for AGP extraction, five kinds of DES (DES-1, DES-2, DES-3, DES-4, and DES-5) based TPP system on the yield of AGP was evaluated with the *t-*butanol-based TPP system as the control. According to the standard curve of glucose shown in Fig. S1 (y = 4.4811x + 0.2439, R^2^ = 0.9985), it was found that the AGP yields in the UA-TPP-DESs group were mainly distributed between 20–25 % (Fig. 1A), which was significantly higher than that of the UA-TPP group (14.38 %). These results indicate that DES is a more effective solvent than *t-*butanol to form a TPP system for AGP extraction, which corresponded with the earlier findings [23]. In addition, by comparing the DES-based TPP extraction system, we found that the TPP system formed by lauric acid and nonanoic acid (molar ratio = 1: 1; DES-4) was more suitable for the extraction of AGP (yield of 24.95 %). Thus, the TPP system formed by DES-4 (UA-TPP-DES-4) was further used in the following experiments.

### 3.2 Signal factor analysis results

The TPP procedure is complex, and influenced by various variables, including extraction time, extraction temperature, the content of (NH_4_)_2_SO_4_, liquid–solid ratio, and the volume ratio of the top phase to the bottom phase [23]. For extraction time, under certain conditions, the yield of AGP-DES-4 was increased with the extraction time increased from 10 to 20 min, while it decreased beyond 20 min (Fig. 1B). The phenomenon might be due to the fact that the dissolution impurities will also increase as extraction time proceeds, finally affecting the AGP extraction yield [27]. Moreover, the yield of AGP-DES-4 reaches a maximum value (16.68 %) under an extraction duration of 20 min. Therefore, the extraction time in the following studies was set as 20 min to ensure the best extraction effect.

In addition to the extraction time, the temperature for extraction was assessed at the specific range of 30 to 70 °C. As suggested by Fig. 1C, the yield of AGP-DES-4 initially enhanced, then receded as extraction temperature rose. It was reported that with heightened temperatures, TPP molecule motion is enhanced, facilitating hydroxyl group exposure, stimulating hydrogen bond inauguration and escalating byproducts' hydrophilicity. This facilitates AGP molecule penetration into the base phase, thereby augmenting AGP-DES-4 extraction yields [14]. However, high-temperature extraction not only enhances the solubility of AGP but also increases the content of water-soluble proteins, thereby affecting the yield of AGP-DES-4. Additionally, high temperature hinders the cavitation of the TPP process, which will also reduce the yield of AGP-DES-4 [14]. Thus, as extraction temperature rises, AGP-DES-4 yield undergoes an initial ascent, followed by a subsequent decrease. Moreover, the yield of AGP-DES-4 reached the highest (13.54 %) when the temperature was 60 °C, and this temperature was selected for subsequent experiments.

According to previous studies, mass fraction of (NH_4_)_2_SO_4_ plays a crucial role in TPP. As suggested by Fig. 1D, the extraction yield of AGP-DES-4 was first increased and then decreased with increase in the mass fraction of (NH_4_)_2_SO_4_. NH_4_^+^ and SO_4_^2-^ could effectively stabilize the interactions between macromolecules, and with the increase in the mass fraction of (NH_4_)_2_SO_4_, the TPP extraction system became more stable, and the polysaccharide became easier to solubilize in the solution. When the mass fraction of (NH_4_)_2_SO_4_ exceeded a specific value, a pronounced salting-out effect will be generated and less water is free to dissolve AGP, consequently resulting in an increased dissolution of AGP in the top or middle phase [28]. The yield of AGP-DES-4 reached the maximum (12.27 %) at 10 % wt (NH_4_)_2_SO_4_, and this mass fraction was selected for the optimal content for the next experiment.

The solid–liquid ratio is another key factor that affecting the yield of AGP-DES-4. As shown in Fig. 1E, when the solid–liquid ratio altered from 1: 10 to 1: 50, the yield of AGP-DES-4 increased first and then decreased. The maximum extraction yield (7.81 %) for AGP-DES-4 was achieved at a solid-to-liquid ratio of 1:20. An increase of the extractant volume increases the osmotic pressure difference between the plant cells and the external solvent, thereby accelerating the diffusion process of AGP from the cells into the solvent, ultimately improving the extraction rate of polysaccharides. However, as the solid–liquid ratio continues to rise, the extraction rate begins to decrease. This phenomenon may be due to excessive addition of solvents causing a large osmotic pressure difference between inside and outside the cells, which in turn leads to the leakage of other cell contents besides polysaccharides, further weakening the concentration difference between inside and outside the cell and ultimately reducing the efficiency of polysaccharide extraction [23]. Thus, the ideal liquid ratio of 1: 20 was identified for subsequent experimentation.

The effect of the volume ratio of the top phase to the bottom phase was also studied. When the volume ratio of the top phase to the bottom phase increased from 1: 0.25 to 1: 2, we found that the extraction yield of AGP-DES-4 was first increased and then decreased. When the volume ratio of the top phase to the bottom phase was 1: 0.5, the extraction yield of AGP-DES-4 reached the maximum, which was 14.76 % (Fig. 1F). DES has similar properties to *t-*butanol in TPP system, it can enhance the buoyancy of proteins and other macromolecules, allowing them to separate from the aqueous phase while dissolving impurities such as lipids and pigments. Insufficient DES can lead to a low polysaccharide extraction yield because DES cannot fully exert a synergistic effect with (NH_4_)_2_SO_4_ [29]. In addition, if the amount of DES is excessive, the extraction yield of AGP could also be affected since the viscosity of the solution will increase, which may reduce the molecular migration rate. Thus, the volume ratio of the top phase to the bottom phase of 1:1 was selected as optimal condition in this study.

### 3.3 Statistical analysis and model fitting

Based on the results of singel factor experiment, BBD experiment was further conducted to optimize the extraction condition of AGP-DES-4. In this BBD experiment, the liquid–solid ratio (A), volume ratio of the top phase to the bottom phase (B), and mass fraction of (NH_4_)_2_SO_4_ (C) were set as independent variables at three different levels (1, 0, −1), while the extraction yield of AGP-DES-4 was used as the response variable for multiple regression fitting analysis. The multiple regression fitting analysis result was shown in Table 3, and the corresponding second order polynomial regression equation was shown as follows.

$$Y=34.58-4.63\times A-1.75\times B-0.1603\times C-0.3328\times AB+0.1525\times AC+0.1784\times BC-13.03{\times A}^{2}-3.32\times B^{2}-2.03{\times C}^{2}$$

**Table 3:** Box-Behnken response surface design and corresponding response values.

Run	Liquid-solid ratio (g/mL)	Top phase to the bottom phase ratio (v/v)	Mass fraction of (NH_4_)_2_SO_4_ (wt%)	Yield (%)
1	10	0.25	10	23.26
2	30	0.25	10	15.69
3	10	1	10	21.7
4	30	1	10	12.29
5	10	0.5	5	24.87
6	30	0.5	5	14.77
7	10	0.5	15	24.39
8	30	0.5	15	14.9
9	20	0.25	5	32
10	20	1	5	27.05
11	20	0.25	15	30.97
12	20	1	15	26.91
13	20	0.5	10	34.83
14	20	0.5	10	35.06
15	20	0.5	10	33.89
16	20	0.5	10	35.05
17	20	0.5	10	35.16

As shown in Table 4, the P value of the regression model is smaller than 0.0001, and the value of the lack of fit is 0.0997, which is higher than 0.05, suggesting that the model adequately fits the experimental data with low error and is less affected by unknown factors. Additionally, as suggested by Table S1, the multiple correlation coefficient (R^2^) was calculated to be 0.996, indicating that there is a good correlation between experimental response values and predicted response values. The Predicted R^2^ of 0.9368 is larger than 0.05 and in reasonable agreement with the Adjusted R^2^ of 0.9899 (difference less than 0.2) indicating that this model has a good fitting effect. The coefficient of variation (C.V. %) was calculated to be 3.084, indicating that the reliability and accuracy of the experiment are relatively high. The P-value not only gauges the importance of each coefficient but further elucidates the interplay of the independent variables. According to the P value shown in Table 4, it was found that the quadratic term (A^2^) for liquid–solid ratio, (B^2^) for top phase to the bottom phase ratio, and (C^2^) for mass fraction of (NH_4_)_2_SO_4_ all reached a highly significant level. Moreover, according to the F-value, it can draw the conclusion that the order of influence of each factor on the extraction yield of AGP-DES-4 was liquid–solid ratio (A) > top phase to the bottom phase ratio (B) > mass fraction of (NH_4_)_2_SO_4_ (C).

**Table 4:** Regression model analysis of variance.

Source	Sum of Squares	df	Mean Square	F-value	P-value	Significant
Model	1019.64	9	113.29	175.57	<0.0001	***
A	162.69	1	162.69	252.12	<0.0001	***
B	24.4	1	24.4	37.8	0.0005	***
C	0.1952	1	0.1952	0.3025	0.5994	n.s.
AB	0.4675	1	0.4675	0.7245	0.4228	n.s.
AC	0.093	1	0.093	0.1442	0.7154	n.s.
BC	0.1344	1	0.1344	0.2083	0.6619	n.s.
A^2^	715.03	1	715.03	1108.08	<0.0001	***
B^2^	34.6	1	34.6	53.63	0.0002	***
C^2^	17.42	1	17.42	26.99	0.0013	***
Residual	4.52	7	0.6453			
Lack of fit	3.43	3	1.14	4.2	0.0997	n.s.
Pure Error	1.09	4	0.2722			
Cor Total	1024.16	16				

*** referred to highly significant (*P* < 0.001); n.s. referred to no significant.

### 3.4 Interaction analysis of each factor in the model

**Fig. 2:**
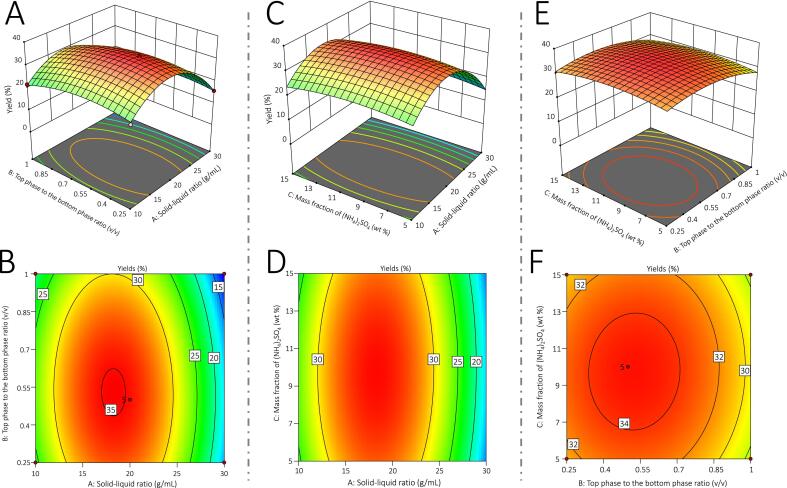
Response surface diagram and contour diagram of the interaction of various experimental factors. (A-B) Interaction between liquid–solid ratio and top phase to the bottom phase ratio; (C-D) Interaction between liquid–solid ratio and mass fraction of (NH_4_)_2_SO_4_ ; (E-F) Interaction between top phase to the bottom phase ratio and mass fraction of (NH_4_)_2_ SO_4_.

The relationships between factor interaction and the extraction yield of AGP-DES-4 were further predicted using 3D response surface. As depicted in Fig. 2A–D, the interaction term of liquid–solid ratio and top phase to the bottom phase ratio (AB) and the interaction term of liquid–solid ratio and mass fraction of (NH_4_)_2_SO_4_ (AC) exhibit an elevated angle of inclination, steep gradient, and elliptical boundary, indicating that both the interaction term of liquid–solid ratio and top phase to the bottom phase ratio (AB) and the interaction term of liquid–solid ratio and mass fraction of (NH_4_)_2_SO_4_ (AC) have a significant effect on the extraction yield of AGP-DES-4. On the contrary, the interaction term of top phase to the bottom phase ratio and mass fraction of (NH_4_)_2_SO_4_ (BC) showed a relatively gentle response surface and an elliptical contour line in Fig. 2E–F, indicating that the interaction term of top phase to the bottom phase ratio and mass fraction of (NH_4_)_2_SO_4_ (BC) had minimal impact on the extraction yield of AGP-DES-4.

The optimal AGP-DES-4 extracted conditions were further predicted by software as follows. Ultrasonic time: 20 min, ultrasonic temperature: 60 °C, ultrasonic power: 100 W, molar ratio of lauric acid and nonanoic acid = 1: 1, liquid–solid ratio = 1: 18.1836, volume ratio of the top phase to the bottom phase = 1: 0.492131, mass fraction of (NH_4_)_2_SO_4_ = 9.33953 % wt. The predicted extraction yield of AGP-DES-4 was 35.1735 %. The actual experimental conditions were set as follows. Ultrasonic time: 20 min, ultrasonic temperature: 60 °C, ultrasonic power: 100 W, molar ratio of lauric acid and nonanoic acid = 1: 1, liquid–solid ratio = 1: 20, volume ratio of the top phase to the bottom phase = 1: 0.5, mass fraction of (NH_4_)_2_SO_4_ = 10 % wt. The actual extraction yield of AGP-DES-4 was calculated to be 35.28 %, which was almost the same as the predicted value, indicating that proposed model was accurate and reliable.

### 3.5 Recycling and reusing of DES

The selected DES was recycled and reused, and the AGP-DES-4 yield was used to evaluate the cycle stability and retrievability of the selected DES. As shown in Fig. S1, after the first recycling, the yield of AGP-DES-4 was calculated to be 35.3 %, which is almost the same as the yield of AGP-DES-4 in the first extraction. As cycling increased, the yield of AGP-DES-4 diminished progressively. When the cycle number reached up to 5, the yield of AGP-DES-4 remained above 30 %, indicating that the DES used in the TPP system had good cycle stability and retrievability.

### 3.6 Structural characterization

#### 3.6.1 Relative molecular weight distribution

The results of molecular weight distributions of AGP-DES-4 were presented in Fig. S2. There was a shoulder peak near *t*_R_ = 11.941 min (3.14 %) and a strong peak near *t*_R_ = 16.807 min (96.86 %) in Fig. S2. Based on the regression equation (y = -0.3382x + 9.2806, R^2^ = 0.9997) derived from the molecular weight standard, the relative molecular weight of AGP-DES-4 ranged from 2.48453 kDa-174.64405 kDa. Recent studies have reported three *American ginseng* polysaccharides with different molecular weight, such as AGBP-A (*M_w_*: 122.988 kDa) [30], PPQN (*M_w_*: 3.1 kDa) [31], and AGP-A (*M_w_*: 5.561 kDa) [32]. The above data demonstrate that the relative molecular weight of AGP-DES-4 includes those of the reported three polysaccharides from *American ginseng,* which indicates AGP-DES-4 may contain the reported three polysaccharides*.* Thus, the extraction technology of AGP-DES-4 could be efficient in the research of *American ginseng* polysaccharides.

#### 3.6.2 FT–IR analysis

**Fig. 3:**
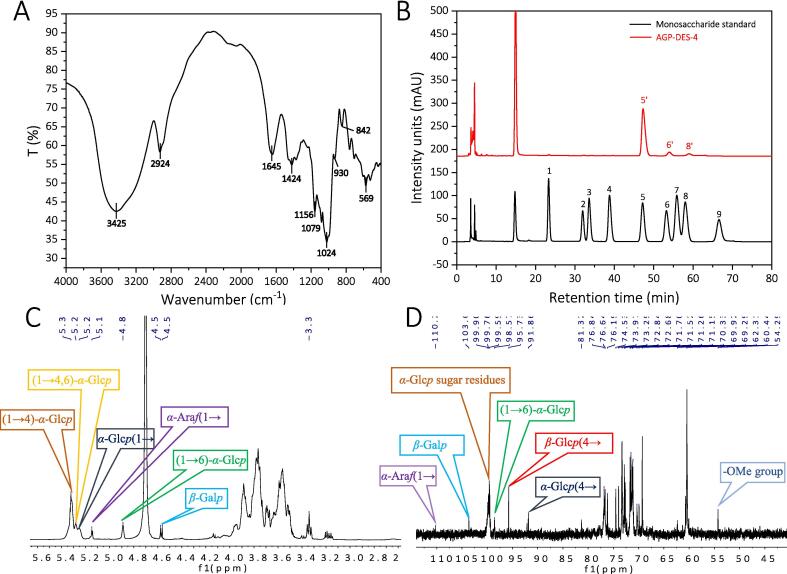
The FT–IR, monosaccharide composition, and NMR spectrum of AGP-DES-4. (A) FT–IR spectra of AGP-DES-4. (B) HPLC profile of the PMP derivatives of monosaccharides from AGP-DES-4. (C-D) ^1^ H and ^13^ C NMR spectra of AGP-DES-4.

Fig. 3A shows the FT-IR spectrum of AGP-DES-4. The prominent peak at 3425 cm^−1^ is due to the O-H stretching of hydroxyl groups, indicating hydrogen bonding within the polysaccharide [33]. A relatively weak band at 2924 cm^−1^ is attributed to the C–H bending vibration [34]. The band at 1645 cm^−1^ represents bound water [35], while the peak at 1424 cm^−1^ denotes C–H bond bending. Three peaks in the 1000–1200 cm^−1^ region indicate asymmetric stretching of C–O–C and C–O–H groups in the pyranose ring [36]. Characteristic absorptions around 930 and 842 cm^−1^ suggest the coexistence of *α* and *β* configurations [31]. Finally, the peak at 569 cm^−1^ signifies the presence of pyran sugar rings [37].

#### 3.6.3 Monosaccharide composition analysis

The monosaccharide composition of AGP-DES-4 was determined utilizing PMP pre-column derivatization. The analysis revealed that AGP-DES-4 primarily consists of glucose, galactose, and arabinose (Fig. 3B). In addition, the content of glucose, galactose, and arabinose in AGP-DES-4 was further quantified using monosaccharide standards (Fig. S3). The result showed that the content of glucose, galactose, and arabinose in AGP-DES-4 was 1.072 mg/mL, 0.063 mg/mL, and 0.02 mg/mL, respectively. This result closely matches the monosaccharide compositions of the reported *American ginseng* polysaccharides, such as AGBP-A (composed of galactose and arabinose) [30], PPQN (composed of glucose and galactose) [31], and AGP-A (composed of glucose) [32].

#### 3.6.4 NMR analysis

The ^1^H and ^13^C NMR spectra of AGP-DES-4 were further used to characterize the structural features of AGP-DES-4. As suggested by Fig. 3C–D, a group of anomeric proton signals ranging from 4.50 to 5.35 ppm was observed in the ^1^H NMR spectrum, while a group of anomeric carbon signals ranging from 91 to 110 ppm was observed in the ^13^C NMR spectrum, indicating that AGP-DES-4 is a heteropolysaccharide. These results were consistent with the results of monosaccharide analysis. Especially, according to the previously reported data, the anomeric proton and anomeric carbon signals of → 4)-*α*-Glc*p*-(1 → were at 5.33/99.6 ppm [38], [39]. Thus, the strongest signals at 5.32 ppm and 99.6 ppm were assigned to the anomeric proton and anomeric carbon signals of → 4)-*α*-Glc*p*-(1 → . Additionally, the signals at 5.28/99.9 ppm might be the anomeric proton and anomeric carbon signals of → 4, 6)-*α*-Glc*p*-(1→ [38], [39]. The signals at 4.89/98.5 ppm might be the anomeric proton and anomeric carbon signals of → 6)-*α*-Glc*p*-(1→ [40], [41], [42], [43]. The signal at 5.25/99.7 ppm might be caused by the anomeric proton and anomeric carbon signals of *α*-Glc*p*-(1→ [44].

In addition, the signals at 5.15 ppm and 110.2 ppm could be assigned to the anomeric proton and anomeric carbon signals of *α*-Ara*f* sugar residues, whose anomeric proton and anomeric carbon signals are mainly distributed in the ranges of 5.10–5.19 ppm and 106–110 ppm, respectively [40], [41], [43]. The signals at 4.56 (4.58)/103.6 ppm might be the anomeric proton and anomeric carbon signals of *β*-Gal*p* sugar residues, which are reported to have their anomeric proton and anomeric carbon signals mainly distributed in the ranges of 4.40–4.70 ppm and 102–104.0 ppm, respectively [45], [46], [47].

Moreover, the signals at 91.9 ppm might be the anomeric carbon signal of *α*-Glc*p* (4 → residue [48], [49]. The 95.7 ppm might be the anomeric carbon signal of *β*-Glc*p* (4 → residue. In addition, the signal at 3.34/54.3 ppm were the typical signals caused by the –OCH_3_ group, indicating that AGP-DES-4 contained –OCH_3_ groups [39], [50].

#### 3.6.5 Congo red analysis

The Congo red assay was used to determine the presence of triple-helical structure in AGP-DES-4. It was reported that within the appropriate concentration range of sodium hydroxide, the Congo red reagent could interact with those triple-helix polysaccharides, causing red shift of the maximum absorption wavelength (*λ*_max_) of Congo red. When the concentration of NaOH exceeded a certain range, the interaction between Congo red and polysaccharide would be broken, leading to a significant decrease of the *λ*_max_ of Congo red. As suggested by Fig. S4, the *λ*_max_ of the AGP-DES-4-Congo red complex does not rise first and then decline, indicating that AGP-DES-4 does not have a triple-helix structure.

### 3.7 *In vivo* anti ulcerative colitis evaluation

The digestive system of *Drosophila* consists of the foregut, anterior ventricle, midgut, and hindgut, with intestinal functions that closely parallel those of mammals [51]. Comparative genomics has revealed that *Drosophila* and humans share 75 % homology in disease-related genes, thus, making *Drosophila* an effective platform for studying gut physiology and pathology. To evaluate the anti-UC activity of AGP-DES-4, a DSS-induced UC *Drosophila* model was established.

#### 3.7.1 Effect of AGP-DES-4 on the survival rate of UC *Drosophila*

**Fig. 4:**
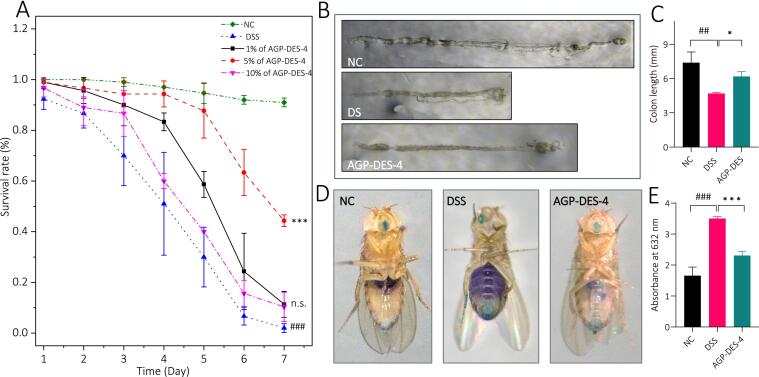
Effects of AGP-DES-4 on the survival rate, intestinal morphology, and intestinal barrier function of UC *Drosophila*. (A) Effects of AGP-DES-4 on the survival rate of UC *Drosophila.* Wild type of *w*^1118^*Drosophila* were treated with 5 % sucrose medium, 5 % sucrose medium containing 3 % DSS, and 5 % sucrose medium containing 3 % DSS supplemented with different concentration of AGP-DES-4 (1 %, 5 %, and 10 %). (B) Representative images of *Drosophila* isolated from different groups (NC, DSS, and AGP-DES-4 treated groups). (C) Quantitative analysis result of the intestinal length of *Drosophila* in different groups (NC, DSS, and AGP-DES-4 treated groups). N = 15. (D) Representative images of *Drosophila* in different groups after blue dye feeding assay. (E) Quantitative analysis result of the absorbance at 632 nm in different groups (NC, DSS, and AGP-DES-4 treated groups). N = 15. The results were expressed as mean ± SD. *P < 0.05 *vs.* the DSS group, ^***^P < 0.001 *vs.* the DSS group, ^##^*P* < 0.01 *vs.* the NC group, ^###^*P* < 0.001 *vs.* the NC group.

The effect of AGP-DES-4 on the survival rate of UC *Drosophila* was evaluated. As shown in Fig. 4A, in comparison with the standard control group, the survival rate of *Drosophila* in DSS group was significantly decreased on day 7 (*P* < 0.001), suggesting that the UC *Drosophila* model was established successfully. After being treated with different concentrations of AGP-DES-4 for 7 days, it was observed that 1 % AGP-DES-4 and 10 % AGP-DES-4 could weakly improve the survival rate of UC *Drosophila*, while 5 % AGP-DES-4 could significantly improve the survival rate of UC *Drosophila* (*P* < 0.001). These results indicate that AGP-DES-4 has significant anti-UC activity. In addition, considering that 5 % AGP-DES-4 showed a more promoting effect compared to 1 % and 10 % concentrations, thus 5 % AGP-DES-4 was selected as the best experimental concentration for subsequent anti-colitis experiments in *Drosophila*.

#### 3.7.2 Effect of AGP-DES-4 on the intestinal morphology of UC *Drosophila*

In mammals, ulcerative colitis lesions typically affect the rectum and sigmoid colon, which can disrupt the intestinal barrier, cause intestinal atrophy, and change intestinal morphology [52]. In the present study, in order to explore whether AGP-DES-4 can protect against DSS-induced UC lesions, the intestines of UC *Drosophila* were collected and compared to evaluate the protective effect of AGP-DES-4 on the intestinal atrophy in *Drosophila*. As shown in Fig. 4B–C, compared with NC group, the intestinal length of *Drosophila* in the DSS group was significantly decreased (*P* < 0.01), indicating that the UC *Drosophila* model was successfully established. However, supplementation with 5 % AGP-DES-4 significantly improved the shortened intestinal length caused by DSS stimulation (*P* < 0.05), which was 32.1 % longer than that of DSS group. This result further confirms the intestine protective effect of AGP-DES-4.

#### 3.7.3 Effect of AGP-DES-4 on the intestinal barrier function of UC *Drosophila*

A blue dye feeding assay was implemented to assess intestinal barrier function. The injury of intestinal barrier function will cause the blue dye throughout the whole body of the *Drosophila* [53]. With the purpose to explore the protective effects of AGP-DES-4 on the intestinal barrier function of UC *Drosophila*, the blue dye feeding assay was carried out in the present study. As depicted in Fig. 4D-E, in comparison with the *Drosophila* in the NC group, the abdomen of DSS-treated *Drosophila* shows a large area of blue, suggesting that the DSS disrupted intestinal permeability and the intestinal barrier function of UC *Drosophila*. However, treatment with AGP-DES-4 significantly reduced the blue infiltration in the abdomen of UC *Drosophila*. In addition, further quantified the absorbance of the crushing homogenate of *Drosophila* at 632 nm, we found that the absorbance at the DSS group was 111.03 % higher than that of the NC group (*P* < 0.001), while the absorbance at AGP-DES-4 treated group was 34.10 % lower than that of the DSS group (*P* < 0.001). These results demonstrate that AGP-DES-4 effectively alleviates the intestinal barrier injury caused by DSS.

#### 3.7.4 Effect of AGP-DES-4 on the intestinal stem cells (ISCs) of UC *Drosophila*

**Fig. 5:**
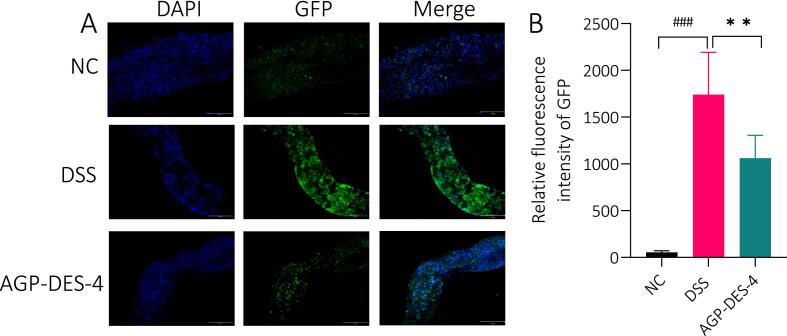
The effect of AGP-DES-4 on the fluorescence intensity of intestinal stem cells in *esg*-Gal4-UAS-GFP/Cyo UC flies. (A) Fluorescence images of intestinal stem cells in the intestines of different groups. (B) Quantitative results of relative fluorescence intensity of GFP-positive cells in the intestines of different groups. Each group consisted of N = 15. Each experiment was repeated three times, and the results were expressed as mean ± SD. ^**^P < 0.01 *vs.* the DSS group, ^###^*P* < 0.001 *vs.* the NC group.

The intestine safeguards against harmful entities ingested via food, functioning as a primary barrier. Intestinal stem cells (ISCs) are among the most active cell populations [54]. Under normal physiological conditions, ISCs proliferate and differentiate into intestinal epithelial cells (IECs) in an orderly manner, preserving gut barrier’s integrity. When the intestinal barrier is disrupted, ISCs continue to proliferate and differentiate, replacing lost or damaged IECs [55]. Therefore, the excessive proliferation and differentiation of ISCs can serve as indicators for observing intestinal barrier damage. To further examine the effect of AGP-DES-4 on ISCs in UC flies , this experiment used transgenic *esg*-Gal4 UAS-GFP/Cyo flies with ISCs labeled with GFP. As shown in Fig. 5A–B, the relative fluorescence intensity significantly increased after DSS induction (*P* < 0.001), reaching more than ten times that of the NC group. This indicates that the DSS disrupted the intestinal barrier, causing a significant proliferation of ISCs, and successfully establishing the UC model. In comparison with the DSS group, the relative fluorescence intensity was significantly reduced under AGP-DES-4 treatment (*P* < 0.01), indicating that AGP-DES-4 can reverse DSS-induced excessive proliferation of ISCs and restore intestinal homeostasis.

#### 3.7.5 Effect of AGP-DES-4 on intestinal epithelial cells (IECs) of UC *Drosophila*

**Fig. 6:**
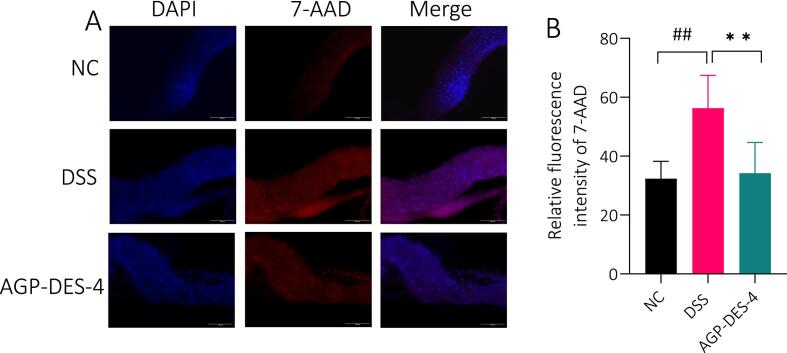
The effect of AGP-DES-4 on the number of intestinal epithelial cells in *esg*-Gal4-UAS-GFP/Cyo UC flies. (A) Fluorescence images of intestinal epithelial cells in the intestines of different groups after staining. (B) Quantitative results of fluorescence intensity after 7-AAD staining of intestinal epithelial cells in different groups. Each group consisted of N = 15. Each experiment was repeated three times, and the results were expressed as mean ± SD. ^**^P < 0.01 *vs.* the DSS group, ^##^*P* < 0.01 *vs.* the NC group.

IECs form the intestinal barrier, tightly arranged on the surface of the intestinal epithelium to protect the body from microbial infections [56]. An abnormal increase in IEC mortality is manifested by epithelial erosion, a feature of several intestinal disorders including inflammatory bowel disease and infectious colitis [57]. Therefore, the number of IECs is an important indicator for evaluating the integrity of the intestinal barrier. In order to investigate the influence of AGP-EDS-4 on UC flies, this experiment used 7-AAD to stain and observe intestinal epithelial cells. 7-AAD is a non-membrane-permeable fluorescent dye that binds to nucleic acids. It cannot penetrate the membranes of living cells but can penetrate those of necrotic cells. Therefore, higher relative fluorescence intensity after 7-AAD staining indicates a higher cell mortality rate. As depicted in Fig. 6A-B, DSS treatment caused a significant increase in fluorescence intensity, compared with the untreated control (*P* < 0.01), reaching approximately twice that of the NC group. This confirmed that DSS successfully induced the UC model. After AGP-EDS-4 treatment, the relative fluorescence intensity significantly decreased to the same level as the NC group (*P* < 0.01), indicating that AGP-EDS-4 can restore DSS-induced damage to IECs and return them to normal levels.

## 4 Conclusion

TPP is promising for isolating and purifying bioactive polysaccharides, but the environmental harm caused by *t*-butanol limits its application. This study optimized an ultrasonic-assisted deep eutectic solvent three-phase partitioning (UA-TPP-DES) system, successfully extracting American ginseng polysaccharides (AGP-DES-4) with high yield and demonstrating that the solvent can be recycled at least five times while maintaining efficiency. Structural analysis and experiments using *Drosophila* models show that AGP-DES-4 has a broad molecular weight distribution and can effectively improve symptoms of ulcerative colitis, highlighting its potential therapeutic value. Although our results provide a new approach for the extraction and purification of AGPs and confirmed that AGP-DES-4 could be therapeutically used as a UC treatment candidate agent, there are still some limitations in this study. These limitations are listed as follows. 1) The optimization process relies heavily on empirical testing, which may not fully capture all variables affecting extraction efficiency. 2) The *in vivo* studies using *Drosophila* models provide promising results, but further validation in mammalian models is necessary to confirm therapeutic efficacy. Future research should focus on expanding the UA-TPP-DES method to other bioactive polysaccharides and optimizing the process for industrial scalability. Additionally, conducting comprehensive clinical trials to evaluate the therapeutic potential of AGP-DES-4 in treating UC will be crucial for translating these findings into practical medical applications.

## CRediT authorship contribution statement

**Zhongnan Wu:** Writing – original draft, Project administration, Data curation. **Chong Li:** Data curation, Formal analysis, Methodology, Project administration. **Junhao Li:** Project administration, Methodology, Investigation, Data curation. **Tanggan Wang:** Writing – original draft, Project administration, Investigation, Data curation. **Meifeng Li:** Project administration, Investigation, Data curation. **Leyi Zhao:** Project administration, Data curation. **Huimei Ye:** Project administration. **Jiaheng Chen:** Project administration. **Jiajia Zan:** Project administration. **Lijun Song:** Conceptualization, Investigation, Supervision, Writing – review & editing. **Qian Zhang:** Project administration. **Shaojie Zhang:** Writing – review & editing, Writing – original draft, Funding acquisition, Conceptualization.

## Declaration of competing interest

The authors declare that they have no known competing financial interests or personal relationships that could have appeared to influence the work reported in this paper.
