# Supplementary material for: Extraction of American ginseng polysaccharide by ultrasound-assisted deep eutectic solvents-based three-phase partitioning: Process optimization, structural characterization, and anti-ulcerative colitis study
Source: Ultrason Sonochem. 2024 Dec 16;112:107206. doi: 10.1016/j.ultsonch.2024.107206 (PMC11732204; doi:10.1016/j.ultsonch.2024.107206)
Supplement: Supplementary Data 1 [file mmc1.docx]

**Supplementary data for**

Extraction of American ginseng polysaccharide by ultrasound-assisted deep eutectic solvents-based three-phase partitioning: Process optimization, structural characterization, and anti-ulcerative colitis study

**Supplementary methods**

***S1 Relative molecular weight distribution of AGP-DES-4***

The relative molecular weight distribution of AGP-DES-4 were determined using an Agilent 1200 high-performance liquid gel permeation (HPGPC) system (Agilent, Santa Clara, CA, USA) equipped with a refractive index detector (RID). The chromatographic column used for detection was a TSK-GEL G3000 PW_XL_ column (Tosoh Biosep, Yamaguchi, Japan). The mobile phase was 0.02 M KH_2_PO_4,_ and the flow rate was 0.5 mL/min. The detection temperature was maintained at 35°C, and the injection volume was 10 *µ*L. In addition, the relative molecular weight distribution of AGP-DES-4 was then determined by establishing a regression equation based on the retention times of the standard pullulans (210000, 113000, 48800, 21700, 2000, and 1000 Da).

***S2 Monosaccharide composition determination of AGP-DES-4***

4 mg of AGP-DES-4 was completely hydrolyzed with 2 M trifluoroacetic acid (TFA) at 120 °C for 6 h. After hydrolysis, the hydrolysate was further co-concentrated with anhydrous methanol four times to remove the TFA. Subsequently, 1 mL of distilled water was added to redissolve the hydrolysate, which was then further reacted with 0.5 M PMP solution (200 *µ*L) and 0.3 M NaOH solution (200 *µ*L) at 70 °C for 30 min. After the reaction, 200 *µ*L of HCl (0.3 M) solution was added to the reaction product. Finally, the reaction products were extracted with chloroform three times to remove the unreacted substances. The aqueous phase was obtained and then analyzed using an HPLC system equipped with a Kromasil 100–5-C18 column (4.6 mm × 250 mm, 5 *μ*m). The chromatographic conditions were set as follows. The mobile phase was a mixture of acetonitrile and a phosphate buffer solution (17:83, v/v, pH = 6.9). The flow rate was 0.8 mL/min, and the detection wavelength was 250 nm.

In addition, the various concentration (0.2, 0.4, 0.6, 0.8, and 1.0 mg/mL) of monoscaccharide standards (Glc, Gal, and Ara) were derivatizated and analyzed using the same method to quantity the content of the monosaccharides in AGP-DES-4.

**Supplementary Tables**

Table S1. Fit statistics analysis

| **Fit Statistics** | | |
| --- | --- | --- |
| 1 | R² | 0.99558950785218 |
| 2 | Adjusted R² | 0.9899188750907 |
| 3 | Predicted R² | 0.93684039188093 |
| 4 | C.V. % | 3.0841034174133 |

**Supplementary Figures**

**Fig. S1** Recycling and reusing analysis of DES. DES was used to re-exrtact American ginseng rots residues for 11 times with the yield of AGP-DES-4 as indicator. Each experiment was repeated three times, and the results were expressed as mean ± SD.

**Fig. S2** HPGPC analysis of AGP-DES-4. (A) HPGPC profile of AGP-DES-4. (B) Standard curve of pullulan for molecular weights calculation. The pullulan standards used are as follows: P-200 : 11.717 min , 210000 Da ; P-100 : 12.453 min , 113000 Da ; P-50 : 13.563 min , 48800 Da ; P-20 : 14.69 min , 21700 Da ; Dextran-2000 : 17.727 min , 2000 Da ; Dextran-1000 : 18.514 min , 1000 Da.

**Fig. S3** The standard curve of Glc, Gal, and Ara used for the conent calculation of the monosaccharides in AGP-DES-4.

**Fig. S4** The *λ*_max_ of the AGP-DES-4-Congo red complex in different concentration of NaOH (0‒0.6 M).
